# Detrended fluctuation analysis of gait dynamics when entraining to music and metronomes at different tempi in persons with multiple sclerosis

**DOI:** 10.1038/s41598-020-69667-8

**Published:** 2020-07-31

**Authors:** Lousin Moumdjian, Pieter-Jan Maes, Simone Dalla Bella, Leslie M. Decker, Bart Moens, Peter Feys, Marc Leman

**Affiliations:** 1 IPEM Institute of Psychoacoustics and Electronic Music, Faculty of Arts and Philosophy, Ghent University, Gent, Belgium; 2 REVAL Rehabilitation Research Center, Faculty of Rehabilitation Sciences, Hasselt University, Hasselt, Belgium; 3 International Laboratory for Brain, Music and Sound Research (BRAMS), Montreal, Canada; 4 Department of Psychology, University of Montreal, Montreal, Canada; 5 Normandie Univ, UNICAEN, INSERM, COMETE, GIP CYCERON, Caen, France; 6 Centre for Research on Brain, Language and Music (CRBLM), Montreal, Canada; 7 University of Economics and Human Sciences in Warsaw, Warsaw, Poland

**Keywords:** Therapeutics, Rehabilitation

## Abstract

In persons with multiple sclerosis (PwMS), synchronizing walking to auditory stimuli such as to music and metronomes have been shown to be feasible, and positive clinical effects have been reported on step frequency and perception of fatigue. Yet, the dynamic interaction during the process of synchronization, such as the coupling of the steps to the beat intervals in music and metronomes, and at different tempi remain unknown. Understanding these interactions are clinically relevant, as it reflects the pattern of step intervals over time, known as gait dynamics. 28 PwMS and 29 healthy controls were instructed to walk to music and metronomes at 6 tempi (0–10% in increments of 2%). Detrended fluctuation analysis was applied to calculate the fractal statistical properties of the gait time-series to quantify gait dynamics by the outcome measure alpha. The results showed no group differences, but significantly higher alpha when walking to music compared to metronomes, and when walking to both stimuli at tempi + 8, + 10% compared to lower tempi. These observations suggest that the precision and adaptation gain differ during the coupling of the steps to beats in music compared to metronomes (continuous compared to discrete auditory structures) and at different tempi (different inter-beat-intervals).

## Introduction

The study of temporal correlations in step or stride intervals over time-also known as gait dynamics^1^- provides useful insights on the neural control of locomotion in young adults^2,3^, healthy older adults^4^, and patients with movement disorders such as Parkinson’s disease^5^, Huntington’s disease^5,6^ or multiple sclerosis (MS)^7^. Gait gives rise to non-stationary inter-step/stride-interval signals, with temporal fluctuations which can be analyzed via non-linear methods^1^. An example of these methods, capable of capturing the complexity of time-evolving behavior in the domain of gait analysis, is detrended fluctuation analysis (DFA). This analysis method can be applied to a time series obtained from gait measurements such as inter-step/stride-intervals^2,3,8^. DFA is robust to non-stationaries in the data, often observed in gait interval time series. This method scales the long-term auto-correlations of non-stationary signals and quantifies the fluctuations in the time series using its self-similar property^8,9^ with a value of fractal scaling index ‘alpha’^8,9^. Alpha provides an estimation of statistical ‘persistence’ or ‘anti-persistence’ in a time series^1^. A healthy value of alpha in gait is between 0.5 and 1.0 (1 being highly persistent), and indicates the presence of statistical persistence within the inter-step-intervals^1,10^. This means that the inter-step-intervals between consecutive steps are non-random and constant at a long range, with small deviations still being present across multiple consecutive strides. On the other hand, a value of alpha < 0.5 signifies the presence of statistical anti-persistence in inter-step-intervals, namely that the inter-step-intervals between consecutive steps are varied and random at a long range^1,10^. In other words, small deviations are immediately corrected on subsequent strides.

Notably, persistent gait dynamics has been associated with a healthy gait, while the loss of persistency in gait dynamics has been associated with aging and neurological disorders such as in Parkinson’s Disease^1^. Loss of statistical persistence in these subgroups has also been related to increased risk of falling^1,4^. However, while these findings may reflect the neural control mechanisms of locomotion, one needs to consider additional mechanisms when interpreting the physiological processes underlying these properties, when gait is modulated by specific task related factors or contexts^11–13^. An important effect of context on the statistical persistence of gait dynamics is observed when walking is entrained to an auditory stimulus. Entrainment is a process that governs the alignment and coupling of the auditory and motor domains such that the period of the steps align with the period or phase of the auditory beat, to reach a state of synchronization^14–16^. This alignment can be understood in terms of coupled oscillators that achieve synchronization by locking into each other's period and/or phase^15,17^, or alternatively, as the effect of minimizing prediction errors^14,15,18^. We therefore hypothesized, that the study of the structure of fluctuations in gait series specifically during a process when the gait is coupled with auditory stimuli would provide a reflection of the underlying predictive processes and timing control engaged during auditory-motor coupling and entrainment.

The structure of fluctuations in the gait time series when walking in time to rhythmic auditory cues has been studied in healthy individuals and patient with Parkinson’s Disease. These studies reported a change in gait dynamics from persistent to anti-persistent behavior when gait was paced by a fixed-tempo metronome as compared to walking without auditory stimuli^19–25^. We assume that this anti-persistent behavior is a reflection of the interaction between the auditory and motor systems during entrainment. Gait dynamics can be modulated by the temporal structure of the stimulus. In healthy controls, the fractal temporal structure of gait dynamics tends toward the statistical properties of the auditory signals^23^. Similar effects were also found in patients with Parkinson’s Disease. Stimuli embedding biological variability (i.e., a metronome with variable inter-beat interval following a fractal structure) resulted in a more persistent gait dynamics in Parkinson’s Disease compared to the isochronous and random-variable (white noise) metronomes^26^. Thus, given that music generally embeds some degree of long-range correlated temporal variability^27,28^ as compared to metronomes, a more persistent gait dynamics should be observed when coupling to music.

In terms of clinical application, the use of music is advantageous to implement in a rehabilitation setting compared to metronomes, as seen in studies with persons with multiple sclerosis (PwMS)^29–31^, a disease where walking impairments are prevalent^32,33^. Additionally, in PwMS, changes in spatiotemporal gait parameters were shown to be dependent on walking impairment and speed instructions^34^. In previous studies, we have shown that PwMS and healthy controls (HC) can sustain auditory-motor coupling^30^ and synchronize their walking to music and metronomes across different tempi^29^. In addition, lower fatigue perception when walking to music compared to metronomes in PwMS compared to HC were reported in these studies. However, little is known about the gait dynamics as a result of the auditory-motor coupling and entrainment when walking to music and metronomes at different tempi. The present study aims to fill this gap in PwMS and HC. That is by applying DFA to investigate the pattern of gait dynamics in PwMS compared to HC, when subjecting the walking to the task-related context of coupling to music and metronomes at different tempi, and in turn understanding the processes underlying auditory-motor entrainment. Notably, gait dynamics measured by DFA was shown not to differ between mildly and moderately impaired PwMS and healthy controls^7^ during walking. Given this evidence, as well as the results from our previous studies in regards to PwMS’s ability to synchronize to music and metronomes^29^, we hypothesize that gait dynamics will not be different between PwMS and HC. We thus assume that both groups will use the same underlying processes to engage in auditory-motor coupling. Rather, we assume that the differences in coupling would be found when entraining to different stimuli (e.g. music and metronomes) and at different tempi.

We hypothesize that walking with music may result in a more persistent gait dynamics compared to walking in time to isochronous metronomes. The type of auditory stimulus (e.g. continuous vs discrete) may influence gait dynamics due to the underlying temporal mechanisms underpinning auditory-motor coupling. In a continuous stimulus such as a sinusoidal pitch change or the complex temporal structure of music, the perceived beat is a property of the stimulus' energetic structure^35^. In contrast, a discrete stimulus includes singular events (e.g., sounds of a metronome) indicating discrete moments in time. Thus, entraining to the continuous structures in music compared to the discrete structures in a metronome is assumed to affect gait dynamics differently. This difference is supported by the theoretical frameworks of emergent and event-based timing^36^ based on discrete and continuous movements^37^, demands on cognition^38^ and by models of the neuronal mechanisms in tracking acoustic events in time, being the cerebellum and the basal-ganglia^39,40^. In addition, we also hypothesize that walking to different tempi would differently influence the gait dynamics. In other words, we assume that the gait dynamics would reflect the process of coupling the footfalls and beats given different inter-beat-intervals presented in the experimental conditions (i.e. presented by the different tempi). This difference is supported by evidence which report that the processing of inter-beat intervals of different durations engage different temporal processing mechanisms^18,36,39,40^.

## Results

28 PwMS (mean age ± SD: 53.45 ± 10.61) and 29 healthy controls (HC) (mean age ± SD: 51.77 ± 11.40) completed the study. PwMS were significantly more impaired than HC on the following motor function tests: the time 25 foot walk (PwMS 7.81 ± 2.11; HC 5.58 ± 0.80 s) and time up and go test (PwMS, 10.94 ± 4.36, HC 6.70 ± 1.17 s).

### Analysis of co-variance

The analysis of co-variance showed no significances between the data-points collected (i.e. the inter-step-intervals) and alpha (F(1,0.002) = 0.09, p = 0.7604, error 0.02). The average and standard deviation of data points collected per participant when walking to music and metronomes a the different tempi are reported in Table 1.

**Table 1:** Average (AVG) and standard deviation (SD) values of the number of data points (i.e. inter-step-intervals), resultant vector length, average baseline speed of participants, and the average speed of the study participants when walking to music and metronomes at different tempi.

	Participants	Walking to music at different tempi						Walking to metronomes at different tempi						
		0	2	4	6	8	10	0	2	4	6	8	10	
Baseline speed (m/s)	HC	1.21 ± 0.13												
	PwMS	0.96 ± 0.23												
Average speed (m/s)	HC	1.16 ± 0.14	1.18 ± 0.15	1.195 ± 0.17	1.19 ± 0.18	1.21 ± 0.15	1.23 ± 0.18	1.18 ± 0.13	1.20 ± 0.15	1.23 ± 0.19	1.26 ± 0.15	1.25 ± 0.18		1.26 ± 0.19
	PwMS	0.91 ± 0.26	0.96 ± 0.27	0.95 ± 0.27	0.96 ± 0.27	0.96 ± 0.27	0.96 ± 0.29	0.92 ± 0.23	0.94 ± 0.25	0.95 ± 0.25	0.97 ± 0.26	1.25 ± 0.18		0.96 ± 0.26
Resultant vector length (0–1)	HC	0.90 ± 0.13	0.87 ± 0.18	0.87 ± 0.16	0.83 ± 0.26	0.80 ± 0.26	0.79 ± 0.23	0.94 ± 0.03	0.94 ± 0.02	0.94 ± 0.03	0.93 ± 0.05	0.90 ± 0.12		0.88 ± 0.17
	PwMS	0.74 ± 0.31	0.77 ± 0.26	0.73 ± 0.31	0.72 ± 0.27	0.70 ± 0.32	0.64 ± 0.34	0.83 ± 0.21	0.79 ± 0.25	0.78 ± 0.27	0.77 ± 0.27	0.77 ± 0.26		0.72 ± 0.31
Inter-step-intervals (number of datapoints)	HC	362 ± 22.42	363 ± 41.85	372 ± 32.40	371 ± 39.83	380 ± 45.80	389 ± 41.19	362 ± 20.79	366 ± 21.77	370 ± 35.53	384 ± 25.91	391 ± 21.08		396 ± 21.70
	PwMS	324 ± 74.99	324 ± 67.29	329 ± 66.82	339 ± 69.87	322 ± 78.15	343 ± 75.00	326 ± 55.01	327 ± 64.78	335 ± 66.13	340 ± 62.77	348 ± 60.8		354 ± 62.30

Study participants comprised of healthy controls (HC) n = 29, persons with multiple sclerosis (PwMS) n = 28.

### Mixed model analysis of variances (ANOVAs)

The experimental results of the below ANOVAs in terms of the *p* and the partial eta squared values are reported in Table 2.

**Table 2:** Experimental results of the main and interaction effects of the statistical mixed model analysis of variance tests and partial eta squared values when walking to music and metronome at different tempi on co-efficient of variation, alpha and resultant vector length.

Outcome measure	Statistical results	Group	Stimuli	Tempi	Assistive device	Baseline speed	Interactions
Co-efficient of variation (%)	P value	ns	0.0007	ns	ns	0.0018	Stimuli*assistive device (p = 0.0054)
	Partial eta squared	–	0.00907	–	–	–	0.00575
Alpha (0–1)	P value	ns	< .0001	< 0.0001	ns	ns	Stimuli*baseline speed(p = 0.0350) ; tempi*baseline speed (p < .0001)
	Partial eta squared	–	0.03377	0.11035	–	–	0.00684; 0.03770
Resultant vector length (0–1)	P value	ns	< .0001	< 0.0001	ns	0.0434	ns
	Partial eta squared	–	0.02266	0.02217	–	–	/
Percentage change in speed (%)	P value	ns	< .0001	< 0.0001	ns	ns	Group*stimuli (p < .0001); stimuli*baseline speed (p < .0001)
	Partial eta squared	–	0.014	0.0036	/	/	0.0467; 0.0237

### Coefficient of variation (CoV) of inter-step-intervals

**Figure 1:**
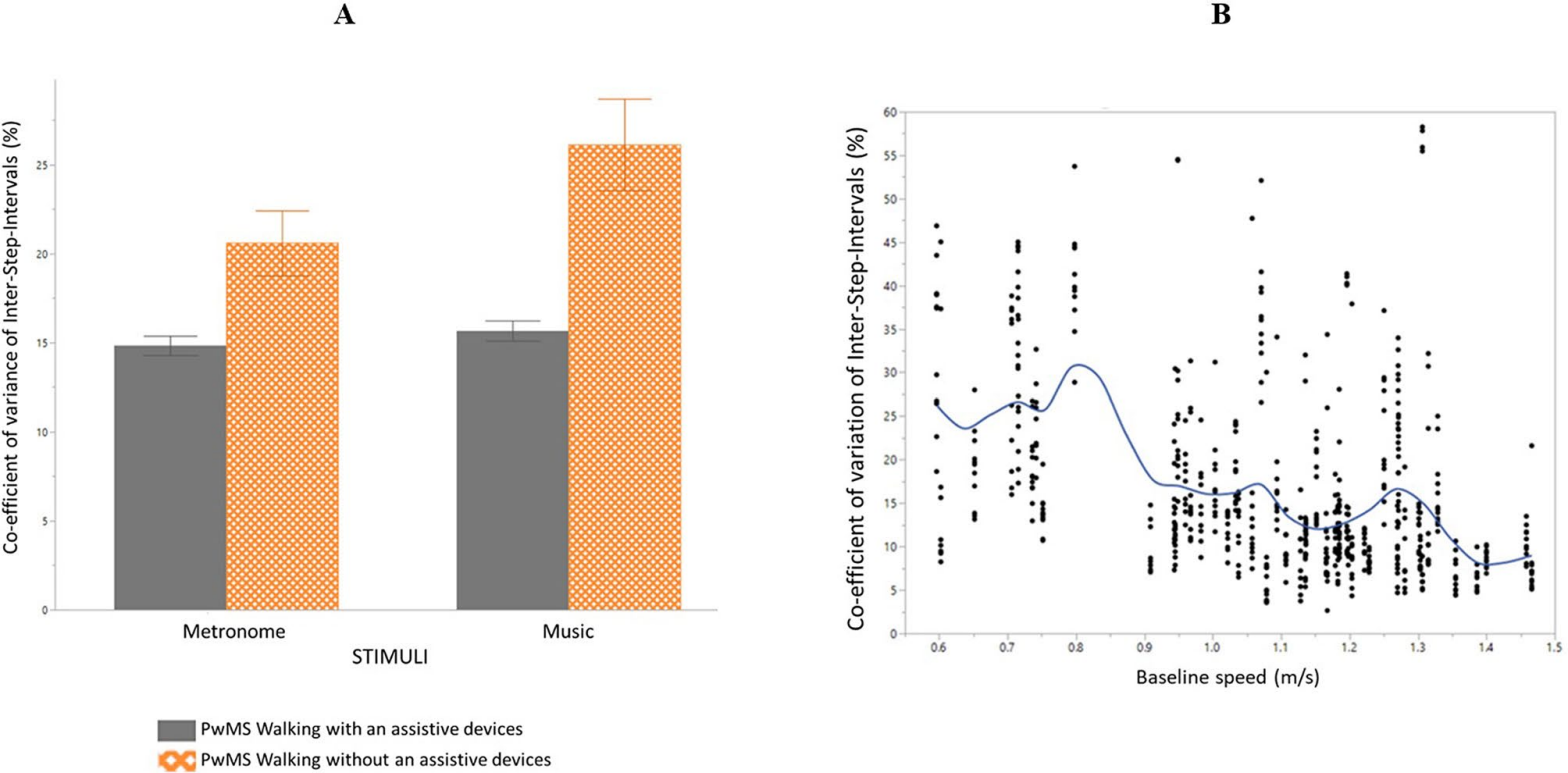
(**A**) Coefficient of variation of inter-step-intervals when waking to a metronomes or music at all experimental tempi in persons with multiple sclerosis (PwMS), divided by patients walking with assistive devices (n = 8) and patients walking without assistive devices (n = 20). Mean and standard errors of mean are shown. (**B**) Co-efficient of variation of inter-step-intervals plotted against baseline speed (m/s) at all experimental tempi in all participants (healthy controls and PwMS) when walking to both music and metronomes.

Significant main effects were found for stimuli (*F*(1,577.8) = 11.5, *p* = 0.0007) and baseline speed (*F*(1,52.3) = 10.8, *p* = 0.0018). The main effect of stimuli indicated a significantly higher CoV when walking to music compared to walking to metronomes (*t* = − 3.39, *p* = 0.0007). A significant interaction effect was found for stimuli*assistive device (*F*(1,577.8) = 7.8, *p* = 0.0054) as seen in Fig. 1A. The post-hoc test indicated significantly higher CoV in PwMS using assistive devices when walking to music compared to walking to metronomes (*t* = − 3.29, *p* = 0.0058). A significant negative correlation was found between baseline speed and CoV, indicating the lower the baseline speed, the higher the CoV (*t* = − 0.43, *p* < 0.0001) as seen in Fig. 1B.

### Fractal dynamics in inter-step-intervals (DFA scaling exponent (α))

**Figure 2:**
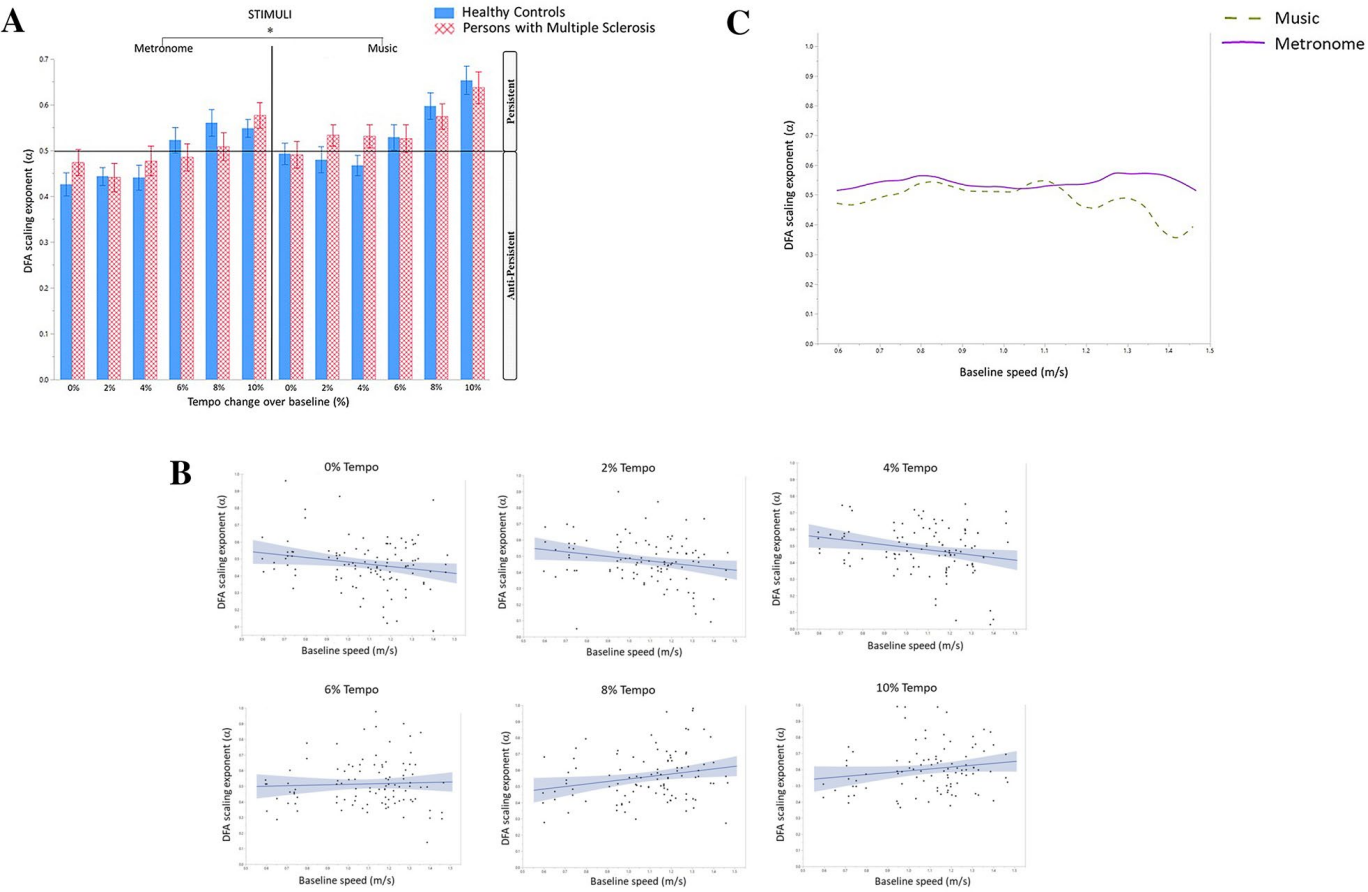
(**A**) The DFA exponent (α) of inter-step-intervals when walking to a metronome or music at different tempi in healthy controls and persons with multiple sclerosis (PwMS). Mean and standard errors of mean are shown. (**B**) The DFA exponent (α) of inter-step-intervals plotted against baseline speed (m/s) at the different experimental tempi in all participants when walking to both music and metronomes. Lines of fit are shown, the figures show a linear regression with confidence intervals for the variables on the x and y axis. (**C**) The DFA exponent (α) of inter-step-intervals plotted against baseline speed (m/s) when walking to music and metronomes for all participants.

Significant main effects were found for stimuli (*F*(1,563.8) = 23.1, *p* < 0.0001) indicating a significantly higher α value when walking to music compared to metronomes, and tempi (*F*(5,557.2) = 16.7, *p* < 0.0001), but no significant effect of group was found as shown in Fig. 2A. Significant interactions were found for tempi*baseline speed (*F*(5,559.2) = 5.4, *p* < 0.0001)) and stimuli*baseline speed (*F*(1,571.8) = 4.5, *p* = 0.0350). The post-hoc tests indicated a significantly higher α value: when walking (i) at the tempo + 8% compared to the tempi + 0, + 2, and + 4% (*t* = − 4.84, *p* < 0.0001; *t* = − 4.75, *p* < 0.0001; *t* =  − 4.37, *p* < 0.0001 respectively), and ii) at the tempo + 10% compared to the tempi + 0, + 2, + 4% and + 6% (*t* =  − 7.05, *p* < 0.0001; *t* =  − 6.95, *p* < 0.0001; *t* =  − 6.58, *p* < 0.0001; *t* =  − 4.55, *p* = 0.0015 respectively). The significant interaction between tempi and baseline speed is shown in Fig. 2B; at higher tempi, persons with higher baseline speed are reported to have higher alpha. The significant interaction between stimuli and baseline speed is shown in Fig. 2C; persons with higher speed are reported to have a higher alpha when walking to music compared to metronomes.

### DFA scaling exponent (α): original vs. surrogate time series

The following effects were found:

(i) A significant effect for time series X^2^(1) = 24.25, p < 0.0001, indicating significantly higher alpha values for the original time series.
(ii) A significant effect for stimuli in the original time series X^2^(1) = 14.41, p = 0.0001, but no significance for stimuli in the surrogate time series X^2^(1) = 3.01, p = 0.00827.
(iii) A significant effect for music in the original time series compared to the surrogate time series X^2^(1) = 29.41, p < 0.0001, but no significance for metronomes between the original and surrogate time series X^2^(1) = 2.38, p = 0.1228.
(iv) No significances for the groups between the original and surrogate time series X^2^(1) = 0.40, p = 0.5377.
(v) A significant effect for tempi in the original time series X^2^(5) = 60.88, p < 0.0001, but no significance for tempi in the surrogate time series X^2^(5) = 1.64, p = 0.8969.
(vi) Significant effects for tempi 8 and 10% between the original and surrogate time series X^2^(1) = 25.23, p < 0.0001; X^2^(1) = 50.71, p < 0.0001 respectively, but no significance for tempi 0, 2, 4 and 6 between the original and surrogate time series X^2^(1) = 0.85, p < 0.3562; X^2^(1) = 0.75, p < 0.3879; X^2^(1) = 0.0005, p < 0.9852; X^2^(1) = 3.54, p < 0.0599 respectively.

**Figure 3:**
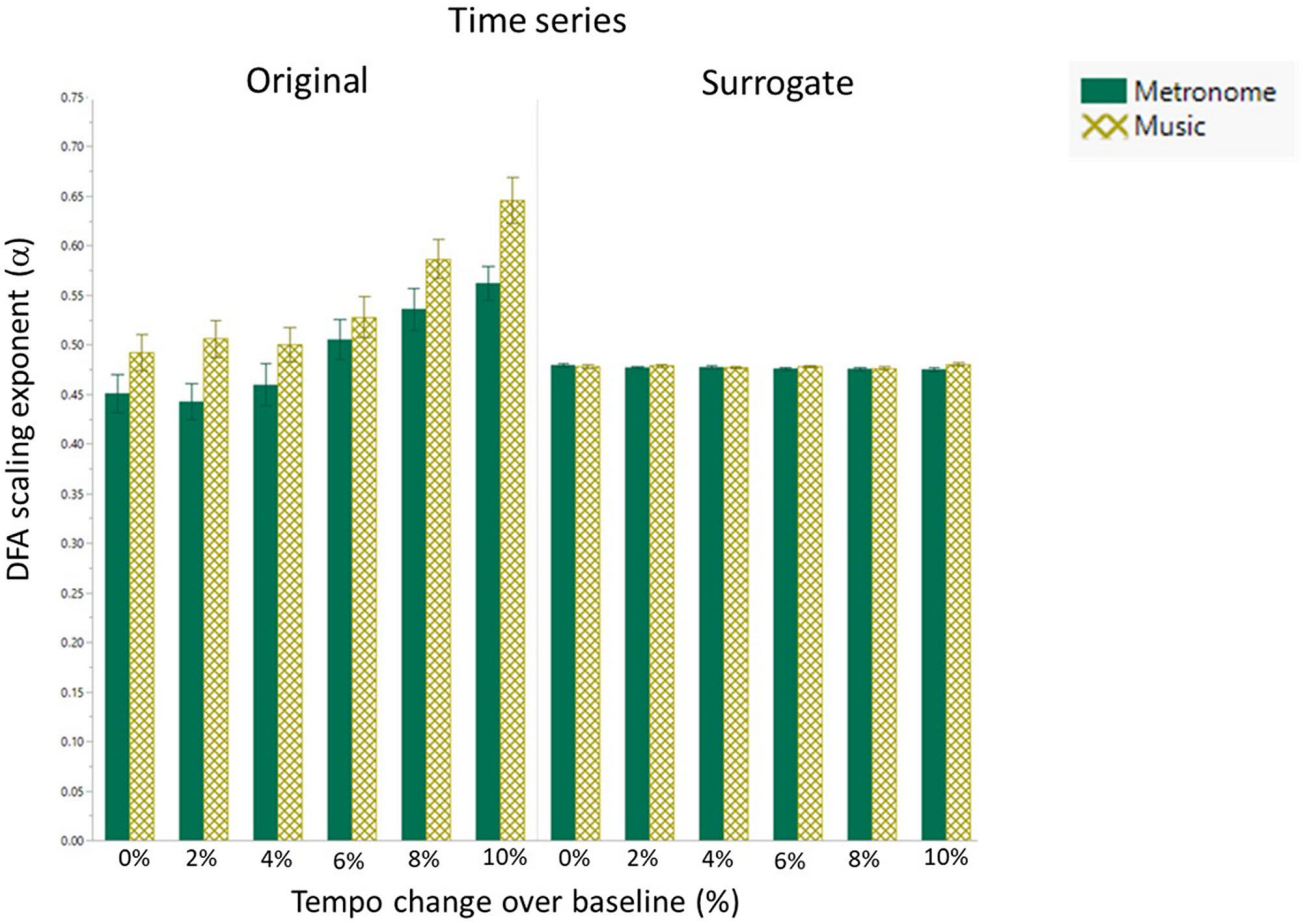
The DFA exponent (α) of the original and surrogate inter-step-intervals time series when walking to a metronome or music at different tempi in healthy controls and persons with multiple sclerosis. Mean and standard errors of mean are shown.

Figure 3 shows graphical representations of these results. Supplementary Fig. 2 additionally provides visualization of the above data in three different perspectives.

### Synchronization consistency

Significant main effects were found for stimuli (*F*(1,579.5) = 27.2, *p* < 0.0001), tempi (*F*(5,579.1) = 5.8, *p* < 0.0001), as previously reported^29^, and baseline speed (*F*(1,51.7) = 4.3, *p* = 0.0434), but no significant effect of group was found. Post-hoc comparisons showed more consistent synchronization: (i) at tempi 0 and + 2% compared to + 10% (*t* = 4.31, *p* = 0.0003; *t* = 4.08, *p* = 0.0007, respectively), and (ii) with a metronome rather than with music (*t* = 5.22, *p* < 0.0001) as seen in Table 1.

### Percentage change in speed

**Figure 4:**
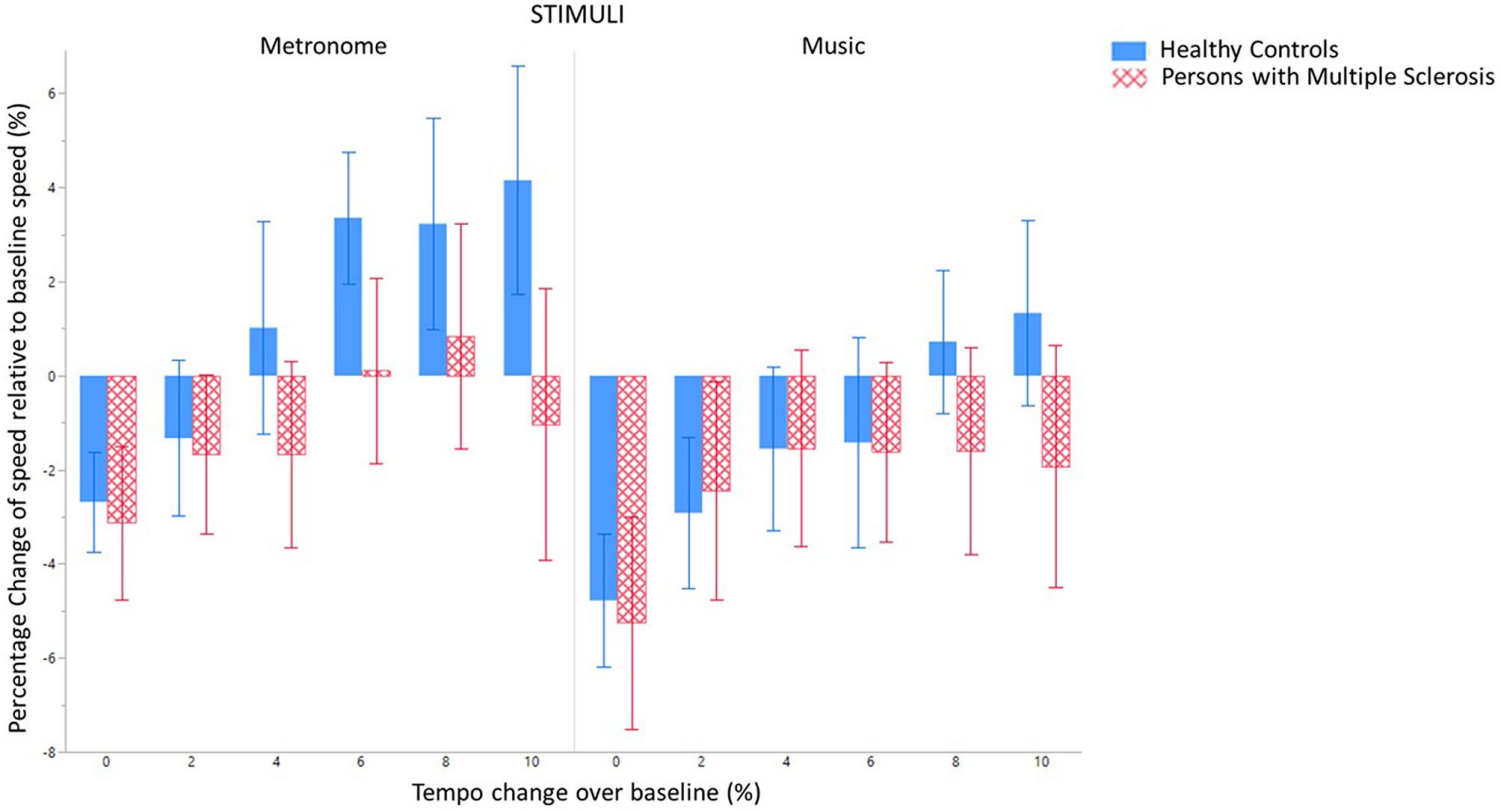
Percentage change in walking speed relative to baseline walking speed when walking to a metronome or music at different tempi in healthy controls and persons with multiple sclerosis. Mean and standard errors of mean are shown.

Significant main effects were found for stimuli (*F*(1,591.1) = 18.9, *p* < 0.0001) and tempi (*F*(5,591.1) = 13.9, *p* < 0.0001), as previously reported^29^, but no significant effect of group was found. Significant interaction effects were found for group*stimuli (*F*(1,591.0) = 16.3, *p* < 0.0001) and stimuli*baseline speed (*F*(1,591.1) = 17.1, *p* < 0.0001). Post-hoc comparisons showed: (i) an increase of speed when walking to metronomes compared to music (*t* = 4.35, *p* < 0.0001), (ii) a lower increase in speed when walking to 0% compared to + 4, + 6, + 8 and + 10% (*t* = − 4.15, *p* = 0.0005; *t* = − 5.87, *p* < 0.0001; *t* = − 6.72, *p* < 0.0001; *t* = − 6.57, *p* < 0.0001, respectively) and 2% compared to + 8 and + 10% (*t* = − 4.03, *p* = 0.0009; *t* = − 3.90, *p* = 0.0015) and (iii) an increase of speed in healthy controls when walking to metronomes compared to walking to music (*t* = 5.97, *p* < 0.0001), as seen in Fig. 4.

Table 1 reports on the average and standard deviation values of the number of data points (i.e. inter-step-intervals), resultant vector length, average baseline speed of participants, and the average speed of participants when walking to music and metronomes at the six different tempi.

### Correlation analysis

No correlations were found between the resultant vector length and the DFA scaling exponent (α) when walking to music or metronomes at all tempi in both HC and PwMS.

## Discussion

In this study, we aimed to understand the underlying process of entraining steps to beats in music and metronomes and at different tempi in PwMS compared to HC. To reach this end, we applied DFA on inter-step-intervals to examine the gait dynamics when PwMS and HC were asked to synchronize their steps to metronomes and music at their preferred walking cadence up to + 10%, in increments of 2%. The analysis of the original and surrogate time series confirmed that the reported statistical persistency measures were not due to chance, but were a reflection of participants’ gait dynamics. In addition, the variability of step duration (coefficient of variation; CoV), synchronization consistency and walking speed were examined.

Studies investigating inter-stride-interval fluctuations reported the presence of anti-persistent gait dynamics as a result of the task-related context. These studies explained that the anti-persistent gait dynamics were observed as a result of deviations in the gait time series caused by participants applying fast corrections to their gait because of the experimental task-related context^12,13^. Similarly, we can interpret our results on gait dynamics in function of the imposed experimental task-related context. Our experimental design provided two manipulations, of the type and of the tempi of the auditory stimulus. The task-related contexts therefore were: coupling to music and metronomes (continuous compared to discrete auditory structures) and coupling to six different tempi (different inter-beat-intervals).

In this study, we found no differences in gait dynamics between PwMS and HC when walking to the auditory stimuli and tempi. To our knowledge, only one study investigated gait dynamics during walking using the DFA method in PwMS with mild and moderate impairment and HC^7^. Albeit having included a small sample (10 persons per group), their results revealed no differences in gait dynamics between PwMS and HC^7^ during walking. Although we cannot directly compare the results of our respective studies due to the different task-related constraint of our walking tasks, their results assist in the interpretation of our results. In other words, when gait dynamics between PwMS and HC are not different at baseline, one can assume that the gait dynamics observed in this study in both groups reflects the auditory-motor coupling process. Our first hypothesis therefore can be endorsed, that both groups engage in auditory-motor coupling using the same underlying processes (explained further in the following paragraphs).

We have however found two patterns of gait dynamics in all participants depending on the auditory stimuli and tempi used during the coupling. Given these differences, our second and third hypotheses can be supported; the auditory-motor coupling processes differ when entraining to music and metronomes and to the different tempi.

First, with metronomes, we found significantly lower alpha values compared to when walking to music, an indication of a trend towards an anti-persistent gait dynamics. This pattern of gait dynamics indicated that the inter-step-intervals between consecutive steps occurred as a non-structured pattern over time. Second, with music, we found significantly higher alpha values compared to when walking to metronomes, indicating a transition to a more persistent gait dynamics. This gait dynamics indicated that the inter-step-intervals between consecutive steps occurred with some structured pattern over time. In addition, when walking to higher tempi to both stimuli, we found a transition to a higher persistent gait dynamics indicated by the significantly higher value of alpha at the + 8% and + 10% as compared to the lower percentages. This gait dynamics indicated that the inter-step-intervals between consecutive steps occurred with more structure over time.

Our results of the trend towards anti-persistent gait dynamics when walking to metronomes are consistent with several studies which reported the same finding when gait was paced by metronomes^19,20,22–25^. Two explanations could be given to our results. The first explanation concerns the presence of variability in inter-beat-intervals in music versus the absence of variability in isochronous metronomes. Earlier evidence presented in several studies on HC and patients with Parkinson’s Disease reported that gait dynamics evolve towards a persistent state once participants get paced with cues that have a random variability^23^, biological variability^26^ and interactive biological variability^41,42^ compared to isochronous metronomes. The second explanation concerns the discrete and continuous energies in the auditory signals. The period of a metronome tick consists of an energy burst followed by a time interval without energy, while the period in music consists of a continuous acoustic energy flow^35^. This energy flow may facilitate the persistence of the gait dynamics through entrainment^43^. The timing mechanisms underlying the temporal processing of the discrete compared to continuous structures in the auditory stream can be referred to as event-based versus emergent timing processes^44^ respectively.

We propose that alpha, as an outcome measure, can explain differences in control mechanisms during the auditory-motor coupling. Below, we provide an explanation for this proposal. During the coupling, a dynamical process (termed entrainment) is engaged through which steps and beats become aligned. The alignment is made possible through prediction error minimization mechanisms. Prediction error here can be seen as the timing differences between steps and beats. Thus, prediction error minimization describes the dynamic process in which the timing differences between steps and the beats become minimized, to reach an alignment. We propose that this dynamic process of prediction error minimization during the coupling is reflected by the gait dynamics^14,15^, measured by alpha. Our results imply that both PwMS and HC engage in this dynamic process of prediction error minimization during the coupling as no group differences were observed in our results.

However, a difference in gait dynamics (alpha) was found between walking to music compared to metronomes. With metronomes, we believe that discrete metronome ticks led to clear-cut attention of errors in step-to-beat alignment^35^ that got instantly corrected in order for alignment to occur^35^. This instant response was reflected in the gait dynamics, and it corresponded to an anti-persistent structure of inter-step-intervals. As the corrections occurred instantly, the intervals had a non-structured (more random) characteristic. This manner of step-to-beat alignment can also explain the significantly higher synchronization constancy when coupling to metronomes, higher walking speed with a lower variability of the step duration.

With music, however, we believe that errors in step-to-beat alignment were less noticeable due to the continues inter-beat-interval structure, making the response to errors less immediate, but more structured over time^35^. This structure was again reflected in the gait dynamics and it corresponded to a persistent structure of inter-step-intervals. The explanations above are supported by existing evidence of different control mechanisms of timing^40^, event based and emergent based control of timing^44^ given the discrete and continuous stimulus structures^35,37,45^. These explanations could also explain our previous behavioral findings showing that walking to music was perceived less cognitively fatiguing than walking to a metronome, even when the coupling was sustained longer with music^30^. This manner of step-to-beat alignment can also explain the significant lower synchronization constancy reported when coupling to music as compared to metronomes, the lower gait speed and higher variability of step durations. It is noteworthy, that the reported level of synchronization consistency when walking to music was adequately high. In addition, the variability of step durations were significantly higher for those participants using an assistive device when walking to music. This finding could indicate that PwMS had a less rigid gait, relying less on the assistive device for movement and more on their own walking when entraining to music compared to metronomes.

Another finding from this study concerns gait dynamics (alpha) at different tempi. One explanation for the differences of alpha observed between the high and low tempi is walking speed, as studies have shown an association between gait speed, gait variability and gait dynamics^46^. Our results also showed that at higher tempi, those participants with higher baseline speeds had higher alpha values. In addition, our results show that the percentage change of speed from baseline was significantly different for the conditions 0% compared to + 4, + 6, + 8 and + 10%, and for condition 2 compared to + 6, + 8 and + 10%. However, it is important to note, that a different pattern was observed by alpha (significantly higher persistency observed at 8 and 10% compared to 0–6% tempi). Furthermore, if the differences of alpha observed were due to walking speed alone, one would expect finding differences in alpha values between those participants with higher baseline speeds and lower baseline speeds across all tempi, while this was not the case. We therefore suggest that walking speed, although an important factor, does not provide a full explanation of our results. Below, we provide a complementary proposal based on the process of prediction error minimization engaged during the coupling.

At higher tempi, we observed a transition to a higher persistent gait dynamics in all participants. At higher tempi, perception of beats becomes more variable. This variance could thereby have an impact on the alignment of steps-to-beats by affecting the individual adaptation processes that persons engage in to achieve step-to-beat alignment. Here, the individual adaptation processes relates to the concepts of precision and adaptation gain in predictive coding^47^. The adaptation gain determines the degree in which the step-to-beat alignment error drives the adaptation towards alignment. The adaptation gain can be defined in terms of a balance between the precision of predicting and performing the step-to-beat alignment versus the precision of perceiving the beat. The adaptation gain could be low if the step-to-beat alignment would have a high precision, while beat perception would have a low precision. In this case, there will be less immediate adaptation to the beat because those noisy perceptions of the beat will be ignored. Consequently, in this case, we propose that the gait dynamics would display a transition towards a more persistent structure, reflecting the fractal structure closer to a natural gait dynamics^1^, not subjected to a task-related context. On the other hand, the adaptation gain could be high if both the step-to-beat alignment and beat perception would have a low precision. In this case the adaptation to the beat would be more immediate, which would display a transition towards a lower persistent gait dynamics. We therefore propose that at higher tempi, HC and PwMS responded with low adaptation gain due to high precision of predicting and performing the step-to-beat alignment, reflected by a transition towards persistent gait dynamics.

With our results, we note that the fractal properties of the inter-step-intervals provided valuable information in regards to timing mechanisms employed during auditory-motor coupling. Specifically, it allowed to interpret the interactive dynamics of a coupled system, whereas gait dynamics had a trend towards anti-persistence when entraining steps to beats metronomes, a trend towards persistence when entraining steps to beats in music, and a trend towards higher persistence when entraining steps to beats of both stimuli at the + 8% and + 10% of preferred walking cadence tempo.

For clinical practice, we recommend to couple steps to beats in music, as the predictive mechanisms engaged when coupling to music resulted in a more persistent and a less rigid gait dynamics when compared to walking to metronomes. In addition, the results of this study contributes to fundamental explanations of the process of coupling walking to music and metronomes at different tempi in viewpoint of predictive coding and timing control. These findings can be used to design, fine-tune, specify and individualize the content of auditory-motor coupling interventions.

Some methodological considerations apply for our experimental design, such as our square (4.5 by 6 m) walking track. We acknowledge that this path involved an environmental constraint, which could have had an effect on alpha^48^. Yet, we believe that this did not have an effect on the comparison of our experimental results because the walking path was standardized for all experimental conditions (i.e. the effect of the environmental constraint was consistent in all experimental conditions). Nevertheless, for future work, we advise over-ground walking conditions with avoidance of 90 degree or 180 sharp turns^48^. A second methodological consideration in this exploratory study is the length of the time series. Obtaining longer time series by increasing the trial durations are advised in future studies. We also advise future studies investigating walking at different tempi to take into account the possible difference in the length of the time series as a function of walking to different tempi (especially when persons are instructed to synchronize). We acknowledge this is a methodological consideration in this current study, and applied transparency by reporting the averages and standard deviations of the length of the time series in our experimental conditions in Table 1. A third methodological consideration is the lack of reporting on the expanded disability status scale due to an incomplete dataset because of the recruitment process.

## Methods

### Participants

30 Persons with Multiple Sclerosis (PwMS) and 30 healthy controls (HC) were included, of which 28 PwMS (mean age ± SD: 53.45 ± 10.61) and 29 HC (mean age ± SD: 51.77 ± 11.40) completed the study. Once recruited, the informed consents were signed and participants were tested for the inclusion criteria, which were: (a) a diagnosis of MS (> 1 year), (b) no exacerbation in the last month, (c) an average comfortable walking speed between 0.4 and 1.4 m per second (m/s), and (d) being older than 18 years of age. Participants were excluded if they were pregnant, had either hearing or cognitive impairment hindering the understanding of instructions. This study is a secondary analysis on a previously reported observational non-blinded case–control study, published in Neuro-Rehabilitation and Neural Repair^29^. The study was approved by the Medical Ethical Committees of universities Hasselt and Ghent (Belgium) and multiple sclerosis centers (The national MS center,Rehabilitation and MS center Overpelt) on November 23rd, 2016 (B670201629797). The study was registered in the clinical trials.gov registry (NCT03281330). All methods were carried out in accordance with relevant guidelines and regulations.

### Materials and experimental procedure

Participants underwent testing in two sessions: in a clinical descriptive testing session, and an experimental testing session, held one week apart. During the descriptive testing session, descriptive demographic and the following clinical motor tests were collected: Time 25 Foot Walk test^49^ for mobility and Time Up and Go test^50^ for balance. During the experimental session, first, participants were asked to walk in their comfort tempo in a square of 4.5 by 6 m three times for one minute, to determine the average preferred walking cadence of the day. This was followed by a familiarization task, using the song ‘Sanctum’ by the artist ‘Shades of the Abyss’ to instruct participants to synchronize by stepping to the beat. This song was chosen because of its clear beats. A similar familiarization was conducted with metronomes as well. Participants then walked three minutes per six tempi to the beat of music and to isochronous metronomes blocks. The tempi were: 0%, + 2%, + 4%, + 6%, + 8%, + 10% of their preferred walking cadence. The tempi and the stimuli blocks were randomized. The D-jogger technology^51^ was used to provide the auditory stimuli at the required conditions. This technology consisted of a software, headphones (Sennheiser, Germany) and two wireless inertial measurement units strapped at the ankles for measuring cadence and step times (iPod, Apple, USA), sampling kinematic gait data at 100 Hz.

### Auditory stimuli

Participants were asked to choose one of six available genres to walk to in the music block from: disco, instrumental, pop, softpop, poprock and variety. See Buhmann et al.^52^ for details of the music data-base generation. Isochronous digital metronomes were used for the metronome block. Supplementary Fig. 1 illustrates the coefficient of variation and standard deviation of the inter-beat-intervals across time of the used stimuli.

### Inter-step-intervals: data processing

Matlab (MathWorks Inc., USA) was used to process the data. The onset of steps logged by the D-jogger system was used to derive step interval time series. The variability of inter-step-intervals was obtained by calculating the coefficient of variation (CoV; a tempo-independent variable) of each time series as a percentage using the following formula: ((standard deviation/mean)*100).

Persistent and anti-persistent structures in step interval time series were examined using Detrended Fluctuation Analysis (DFA). DFA is a method that removes local trends, thus it is less likely to be affected by non-stationarity in the time series^2^. DFA computes the mean square roots of (linearly) detrended residuals, $F(n)$, of the integrated time series over a range of equal, non-overlapping window sizes $n$. The scaling exponent *α* is then estimated from the slope of the linear relationship between $log[F(n)]$ and $log(n)$. Restricted range of window sizes were used, from $n$ = 4 steps to $n$ = $N$/4 steps^53,54^ (N being the total number of steps). An $\alpha$ > 0.5 indicates statistical persistence, with fluctuations in one direction followed by fluctuations in the same direction. An $\alpha$ < 0.5 indicate anti-persistence, with subsequent fluctuations in the opposite direction. An $\alpha$ = 0.5 indicates uncorrelated noise, with subsequent fluctuations equally in either direction. In the context of control, gait variables that are not tightly regulated exhibit strong persistence, while those that are more tightly regulated exhibit either uncorrelated or anti-persistent fluctuations ($\alpha$ ≤ ~ 0.5). Details of the methodology are published elsewhere^2,8,12,55–57^. To determine if relatively simple random processes might account for the statistical properties seen within the experimental data, surrogate time series were generated by randomly shuffling each original time series collected per participant and per experimental condition one hundred times^58,59^. In other words, one hundred surrogate time-series were generated per participant and per experimental condition. Thereafter, per participant and experimental condition, one mean alpha value was calculated from the distribution of the surrogate time series.

### Synchronization with the auditory stimuli

Synchronization to the stimulus beat was measured by calculating the resultant vector length (RVL). The RVL is a measure that indicates the consistency of timing differences between two periodic signals. Here, these were the timing difference of the individual steps relative to their closest beats. These timing differences were calculated and expressed as phase angles. Thereafter, the average of the sine and cosine coordinates of all phase angles were calculated, in order to obtain the RVL^60,61^. The RVL represents how well the participants matched their steps to the beats over time. RVL is expressed as a number from 0 to 1. Higher numbers indicate more consistent synchronization, meaning that the timing differences between individual steps and beats become concentrated around a single value. Lower numbers indicate inconsistency of synchronization, meaning the timing differences between individual steps and beats become highly variable over time. Please refer to a methodological review for details^16^.

### Spatio-temporal gait parameters

Participants were equipped with three OPAL wearable sensors (Mobility lab, APDM, USA), two strapped on their ankles, and one strapped on the sternum, to measure the spatiotemporal gait parameters^62^. Spatio-temporal gait measurements, e.g. cadence and speed were obtained during the baseline walking conditions (when walking without any auditory stimuli) and the experimental walking conditions (when walking to the auditory stimuli). Thereafter, percentage change in speed was calculated as a percentage change between the average speed of the participant at baseline compared to their average speed during the experimental conditions.

### Statistical analysis

Four mixed model analysis of variances (ANOVA) were performed, one on the primary outcome measure (α value) of the original time series and three for secondary outcome measures (RVL, CoV, percentage change in speed). The independent variables were: group (HC and PwMS) as between-subjects factor; stimuli (music and metronome) experimental tempi (0, + 2, + 4, + 6, + 8 and + 10% of the preferred walking cadence) as within-subjects factor. In addition, two co-variates were added to the analysis, these were assistive devices (yes (n = 8) and no (n = 20)) and the baseline walking speed of participants. These co-variates were added to the analysis as confounding variables, as assistive devices and walking speed have shown to effect gait variability^46,63^. Multiple comparisons Tukey’s tests were further performed as post-hoc tests. For the ANOVA’s above, assumptions of normality and homoscedasticity have been verified by visually examining each model’s residual quantile and predicted plots. In addition, six Kruskal–Wallis tests were conducted to statistically examine differences in alpha values between the time series (original and surrogate), stimuli (music and metronome), group (HC and PwMS) and conditions (*0, 2, 4, 6, 8, 10%*). Bonferroni’s correction was applied to correct for multiple comparisons. The following were examined: comparison between the time series; comparison of the time series between stimuli; comparison of the stimuli between the time series; comparison of the groups between the time series; comparison of the tempi between the time series and; comparison of the time series between the tempi. To further examine the relationship between synchronization and α, Spearman’s correlation coefficients were calculated for PwMS and HC when walking to music or metronomes at the six different tempi. All analyses were performed using SAS JMP Pro 13.2.0 (copyright SAS Institute Inc., USA). The significance level was set at *p* < 0.05. Given the experimental design (i.e. the fixed time of walking three minutes per tempi) it was expected for cadence to be modified across the different tempi, and as a result, the number of steps collected (i.e. data points) could differ across tempi. To control correctly for the discrepancy, prior to running the ANOVAs described above, an analysis of covariance was applied by fitting a standard least squares to the outcome alpha by the total number of data points collected per trial.

## Supplementary information

Supplementary information

## Data Availability

The datasets generated during and/or analyzed during the current study are not publicly available due to privacy regulations but are available from the corresponding author on reasonable request until 2022.
