# Supplementary material for: Detrended fluctuation analysis of gait dynamics when entraining to music and metronomes at different tempi in persons with multiple sclerosis
Source: Sci Rep. 2020 Jul 31;10:12934. doi: 10.1038/s41598-020-69667-8 (PMC7395137; doi:10.1038/s41598-020-69667-8)

Title

Detrended fluctuation analysis of gait dynamics when entraining to music and metronomes at different tempi in persons with multiple sclerosis

Authors

Lousin Moumdjian[1,2*], Pieter-Jan Maes[1], Simone Dalla Bella[3,4], Leslie M. Decker[5], Bart Moens[1], Peter Feys[2], Marc Leman[1]

Affiliations

[1]IPEM Institute of Psychoacoustics and Electronic Music, Faculty of Arts and Philosophy, Ghent University, Gent, Belgium

[2] REVAL Rehabilitation Research Center, Faculty of Rehabilitation Sciences, BIOMED, Hasselt University, Hasselt, Belgium

[3] International Laboratory for Brain, Music and Sound Research (BRAMS), Montreal, Canada

[4] Department of Psychology, University of Montreal, Montreal, Canada

[5] Normandie Univ, UNICAEN, INSERM, COMETE, GIP CYCERON, Caen, France.

*Corresponding author

Contact information: Lousin.moumddjian@ugent.be; lousin.moumdjian@uhasselt.be

Supplementary Figure 1. Coefficient of variation and standard deviation of the inter beat interval time series for the six different music genres and metronome.

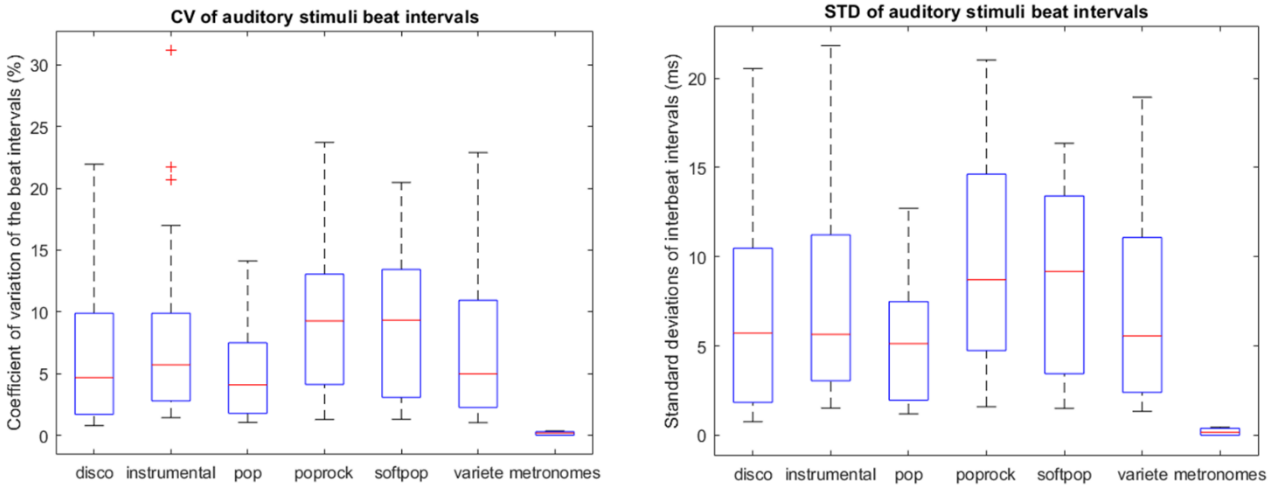

Supplementary Figure 2. DFA exponent (α) of the original and surrogate inter-step-intervals time series. The data is visualized in three different ways. A: scatter plot illustrating the distribution of alpha per participant collapsed per stimuli, in the original and surrogate time series. B: scatter plot illustrating the distribution of alpha per participant per experimental condition and per stimuli, in the original time series and surrogate time series. C: bar plots, illustrating the distribution of alpha per tempi and per stimuli, in the original and surrogate time series (mean and standard errors of mean are shown).

A

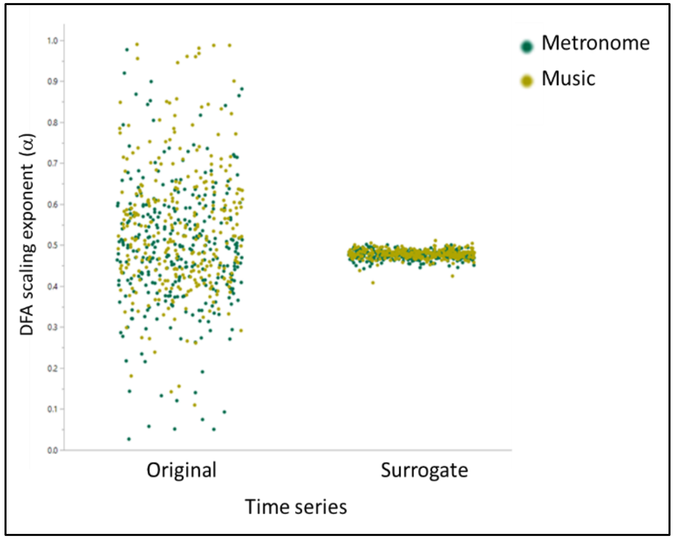

B

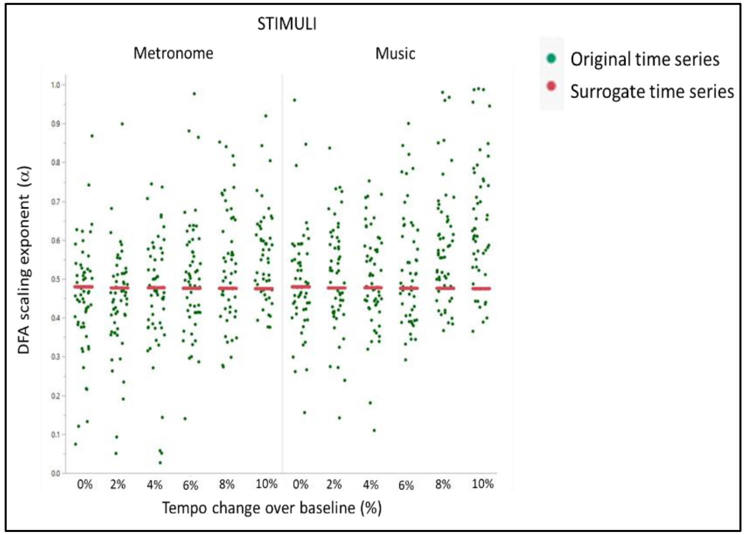

C

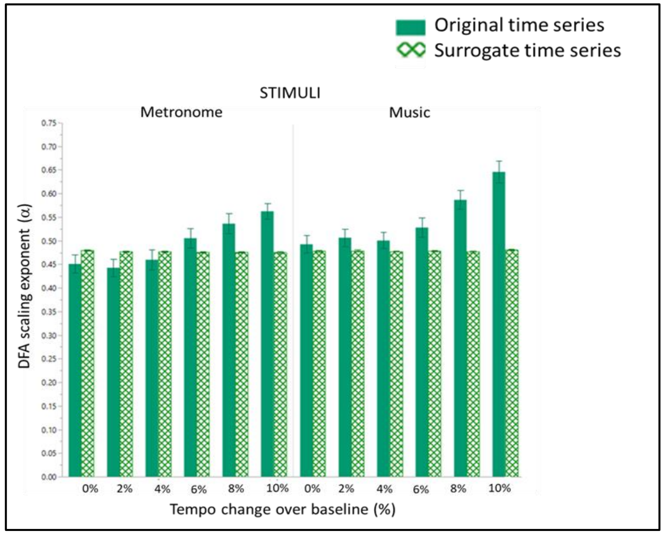

Supplement: Supplementary file 1 — Supplementary information [file 41598_2020_69667_MOESM1_ESM.pdf]
